# Lung squamous cell carcinoma metastasizing to the nasopharynx following bronchoscopy intervention therapies: a case report

**DOI:** 10.1186/1477-7819-12-68

**Published:** 2014-03-27

**Authors:** Jian-bin Hu, Mei Jin, En-guo Chen, Xiao-nan Sun

**Affiliations:** 1 Department of Radiation Oncology, Sir Run Run Shaw Hospital of Zhejiang University Medical School, No. 3 Qingchun East Road, 310016 Hangzhou, Zhejiang Province, China; 2 Department of Pathology, Sir Run Run Shaw Hospital of Zhejiang University Medical School, No. 3 Qingchun East Road, 310016 Hangzhou, Zhejiang Province, China; 3 Department of Respiratory Medicine, Sir Run Run Shaw Hospital of Zhejiang University Medical School, No. 3 Qingchun East Road, 310016 Hangzhou, Zhejiang Province, China

**Keywords:** Lung cancer, Metastasis, Nasopharynx, Squamous cell carcinoma

## Abstract

Metastatic carcinoma to the nasopharynx is extremely rare, and few cases have been reported in the literature. In the present report, we describe the case of a patient with a mass in the nasopharynx found by bronchoscopy. Our patient was a 61-year-old man receiving multiple bronchoscopy intervention therapies for advanced lung squamous cell carcinoma (SCC), which was histopathologically confirmed. The SCC metastasized to the nasopharynx following the bronchoscopy intervention therapies. The lesion was considered metastatic from lung cancer on the basis of clinical and histological clues. The exact mechanism of lung cancer metastasis to the nasopharynx in this case remains unclear because either implantation or hematogenous and lymphatic spread is possible. A thorough head and neck examination should be undertaken during bronchoscopic evaluation, especially in patients receiving bronchoscopy intervention therapies. The early detection of a silent nasopharyngeal metastasis is important to choosing from among the multiple treatment options available.

## Background

Carcinoma metastatic to the nasopharynx is rare, and there are few reports in the literature concerning this condition [1,2]. More specifically, nasopharyngeal metastasis from lung cancer is extremely rare, with very few cases reported to date [3,4]. In this case report, we describe an unusual case of lung squamous cell carcinoma (SCC) that metastasized to the nasopharynx following bronchoscopy intervention therapies.

## Case presentation

**Figure 1:**
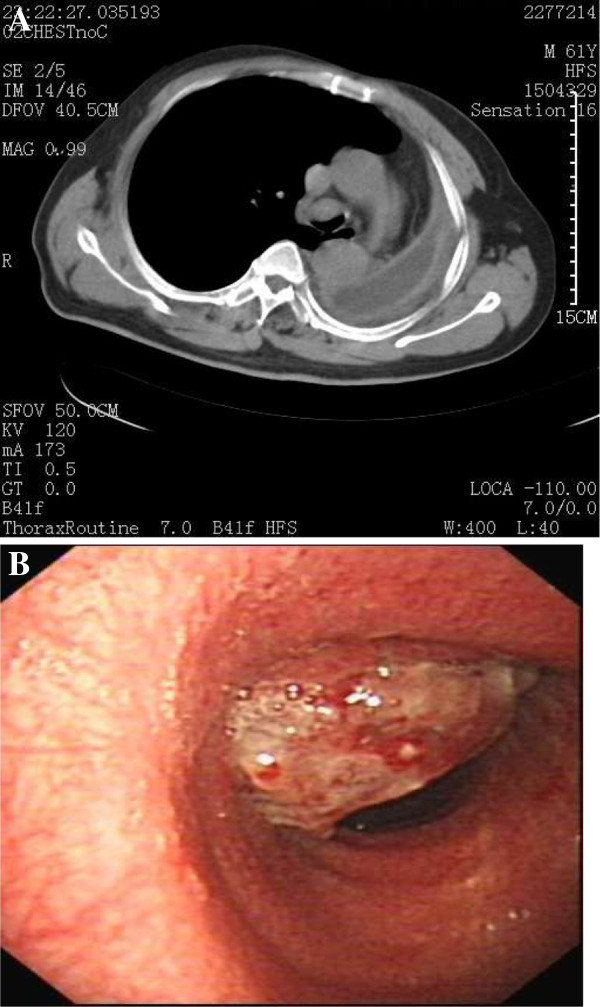
**Scans of the patient’s chest show the mass in the end of the trachea. (A)** Computed tomography scan. **(B)** Bronchoscopy.

**Figure 2:**
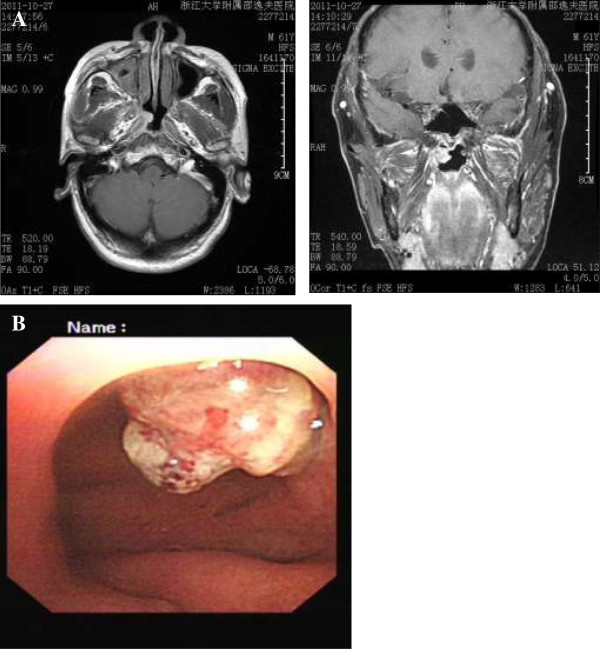
**Scans show the mass in the torus tubarius of the nasopharynx. (A)** Magnetic resonance imaging scan. **(B)** Bronchoscopy.

**Figure 3:**
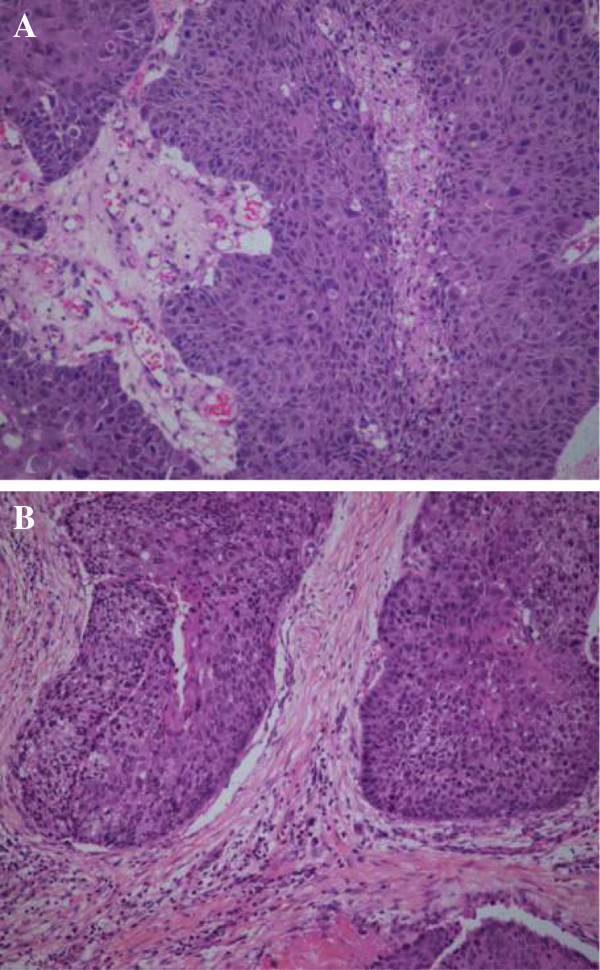
**Histopathological specimen images.** Nasopharynx specimen **(A)** showing squamous cell carcinoma with morphology similar to that of the primary lesion of the trachea **(B)**.

A 61-year-old man who was a heavy cigarette smokerpresented to our department with a mass in the nasopharynx found by bronchoscopy. He had been diagnosed with SCC of the left lower lung by bronchoscopic biopsy 2 years earlier. He was staged with T4N0M0 disease after systemic evaluation at that time. Thereafter he received a presumed curative left pneumonectomy, which revealed moderately differentiated SCC with no lymph node metastases and a clear bronchial resection margin. He was given four cycles of adjuvant chemotherapy with regular doses of gemcitabine and cisplatin. His disease had remained stable for 1 year until 6 months before his presentation to our hospital.We founda recurrent mass in the end of his trachea by chest computed tomography and bronchoscopy (Figure 1), and, on the basis of a bronchoscopic biopsy, we confirmed the mass to be a well- to moderately differentiated SCC. To alleviate the patient’s symptoms of respiratory obstruction, tumor removal was carried out through bronchoscopy intervention therapies, including argon knife, electric snare, cryotherapy and hot biopsy forceps. His symptoms were relieved after these procedures. However, his symptoms recurred 1.5 months later. A similar mass was found again in the end of the trachea, with the same pathological result confirmed by biopsy. The second-course tumor removal through bronchoscopy intervention therapy was carried out, followed by one course of conformal external beam radiation to the tumor bed at a dose of 6,600 cGy in 33 fractions. Three months after the completion of radiotherapy, a cauliflower-like mass in the torus tubarius of the nasopharynx was found during a routine follow-up bronchoscopic evaluation. Biopsy of the nasopharyngeal mass confirmed it to be SCC. The patient was referred to our department for further evaluation. Serum tests of antibodies to Epstein-Barr virus (EBV) were negative. Magnetic resonance imaging revealed a solitary mass in the torus tubarius of the nasopharynx without paranasopharyngeal space involvement (Figure 2). Owing to the patient’s advanced disease and poor general condition, palliative removal of the lesion in the nasopharynx was carried out through bronchoscopy intervention therapy. Histopathological examinationof hematoxylin and eosin–stained sections of the nasopharyngeal lesion revealed typical SCC composed of large, eosinophilic cells with distinct cell borders, keratinization and the formation of small horn pearls. The histopathological morphology of the nasopharyngeal lesion was similar to that of the primary tumor of the trachea (Figure 3). To identify the origin of the lesions, samples of nasopharyngeal and tracheal masses were analyzed for EBV infection status by the technique of EBV-encoded RNA *in situ* hybridization (EBER-ISH). EBER-ISH tests of both lesionswere negative. The patient received an additional five cycles of chemotherapy with regular doses of taxotere and cisplatin. He remained disease-free at his 6-month follow-up examination.

## Discussion

As defined by the World Health Organization classification system, nasopharyngeal carcinoma (NPC) histopathologically can be classified into undifferentiated carcinoma (UC), differentiated nonkeratinizing carcinoma (NKC) and SCC [5]. In the case of our patient, examination of tissues obtained from of the nasopharynx was enough to establish the diagnosis of SCC. Histologically, the tumor shows prominent features of keratinization, including the presence of squamous pearl formation and evident intercellular bridges. Negative serum antibody to EBV and negative EBER-ISH, which is almost invariably positive in UC and NKC and less often positive in SCC, also support the diagnosis [6-8]. However, identification of metastatic or primary nature of nasopharyngeal SCC is difficult and is imperative in making the appropriate prognostic evaluation and choosing the best treatment option. In our patient, a documented previous history of advanced lung SCC associated with recurrence was highly suggestive of the metastatic nature of the lesion in the nasopharynx. However, tumors seldom metastasize to the nasopharynx. In contrast, the incidence of primary lung cancer associated with second primary tumors in the head and neck area is 1% to 2% [9,10]. Because of the very low prevalence of nasopharyngeal metastasis and relatively more common secondary primary tumors, it could be argued that a history of malignancy does not necessarily imply that a nasopharyngeal lesion is metastatic in nature. It is indeed well-known among pathologists that there are currently no immunohistochemical markers for the determination of the likely site of origin of SCC, even though this is possible for adenocarcinomas in most cases [11]. Some relevant and heuristic studies have been carried out in recent years. Kanthan *et al*. adopted cytokeratin CK5, p63 and p16 as immunohistochemical markers to confirm the diagnosis of SCC of the cervix metastatic to the duodenum [12]. Huang *et al*. performed immunohistochemical staining to compare the expression pattern of the epithelial–mesenchymal transition markers between primary SCC of the hypopharynx and a metastatic lesion of the duodenum [13]. Zhang *et al*. identified the *SPLUNC1*and *LPLUNC1* genes by suppression subtractive hybridization and cDNA microarray. These genes are specifically expressed in normal adult nasopharyngeal epithelial tissue and trachea and downexpressed in NPC [14]. Gevaert *et al*. studied the expression profiles of a series of human epithelial cancers and their metastases by microarray technology. Strikingly, they concluded that SCCs do not reflect their primary tissue expression profile because of the absence of a molecular signature in these tumors that reflects their tissue of origin [15]. Therefore, the differentiation between primary and metastasis of nasopharyngeal SCC currently depends mainly on clinical and histological signs. In our region, malignancies in the nasopharynx are largely confined to the pharyngeal recess [16]. Some have argued that the roof of the nasopharyngeal cavity may be the area most likely to develop the original carcinomatous microfocus, followed in frequency by the posterior wall [17]. Early cancers localized to the torus tubarius are rarely reported. Histologically, undifferentiated or NKC is the main subtype, which is associated with the EBV genome within the tumor and intratumoral lymphoid infiltrate. Keratinizing SCC is uncommon and accounts for only about 5% of cases [18]. The clinical clues highly reminiscent of metastasis to the nasopharynx in this case include a documented previous history of advanced lung SCC associated with recurrence, the uncommon location of the lesion, the cauliflower-like tumor growth without paranasopharyngeal space involvement, the absence of enlarged cervical lymph nodes, negative serum antibody to EBV and multiple bronchoscopy intervention therapies. Histological signs suggestive of metastasis include the unusual histological subtype, negative EBER-ISH, lack of local inflammatory response and absence of squamous metaplasia in the mucosa adjacent to the tumor.

The exact mechanism of lung cancer metastatic to the nasopharynx in our patient remains unclear. Significantly, during his treatment, the patient underwent two-course bronchoscopy intervention therapies for obstruction of the airway through his right nostril. Portsite metastasis secondary to instrumentation in laparoscopic surgery has been well-documented. Numerous mechanisms have been proposed, including implantation, leakage of insufflation gas through the ports (the “chimney effect”) and the effect of pneumoperitoneum on local immune reactions [19]. More than 30 cases of metastatic spread to a percutaneous endoscopic gastrostomy site in patients with head and neck cancer have been reported [20], and direct implantation of cells is considered more likely than hematogenous spread [21]. More rarely, cases of nasal tip metastasis from various malignancies have been reported as well [22]. It can be postulated that the metastatic mechanism is similar with the use of such instrumentation. Implantation is the most likely cause. It has been shown that instruments harbor tumor cells and that tissue trauma, tumor manipulation and spillage increase the chances of tumor seeding [23]. Hematogenous and lymphatic spread must also be considered. It is well-known that metastases often favor sites of trauma. It is possible, therefore, that the nasopharyngeal mucosa traumatized by the bronchoscopy in our patient may have attracted blood-borne metastases. This hypothesis is in agreement with our patient’s advanced cancer stage, which implies that tumor cells were circulating in the lymphatic channels and bloodstream.

Because of the extreme rarity of nasopharyngeal metastasis, there is no consensus regarding the best treatment. Wong *et al*. reported the case of a patient with adenocarcinoma of the lung with solitary nasopharyngeal metastasis. Their patient received palliation radiotherapy and remained disease-free for 10 years from the date of diagnosis of nasopharyngeal metastasis, and they postulated that solitary nasopharyngeal metastasis from lung primary tumor might be a separate entity that responds well to radiotherapy [4]. Saab *et al*. reported the case of a patient with breast carcinoma metastatic to the nasopharynx with a dismal result due to delay of treatment [1]. In our present case, we adopted palliative bronchoscopy intervention therapy but not radiotherapy to the nasopharyngeal lesion because of the metastatic nature with implantation as the probable cause, the absence of an enlarged cervical lymph node or paranasopharyngeal space involvement and the advanced primary lung cancer. The long-term result of treatment awaits further evaluation.

## Conclusions

Metastasis to the nasopharynx from primary lung cancer is a rare occurrence. A thorough head and neck examination should be undertaken during bronchoscopic evaluation, especially in patients receiving bronchoscopy intervention therapies. The early detection of a silent nasopharyngeal metastasis is meaningful in choosing from among the multiple treatment options available. Otherwise, such a metastasis could become symptomatic and difficult to control because of its potential to invade the base of the skull.

## Consent

Written informed consent was obtained from the patient’s family for publication of this case report and any accompanying images. A copy of the written consent is available for review by the Editor-in-Chief of this journal.

## Competing interests

The authors declare that they have no competing interests.

## Authors’ contributions

JH and XS operated on the patient and were major contributors to the writing of the manuscript. MJ interpreted the patient’s histological data. EC administered the bronchoscopy intervention therapies. All authors contributed to the intellectual content of the report, and all authors read and approved the final version of the manuscript.
